# Executive function predictors of science achievement in middle-school students

**DOI:** 10.3389/fpsyg.2023.1197002

**Published:** 2023-11-28

**Authors:** Keisha Varma, Martin Van Boekel, Gary Aylward, Sashank Varma

**Affiliations:** ^1^Department of Educational Psychology, University of Minnesota Twin Cities, Minneapolis, MN, United States; ^2^School of Interactive Computing and School of Psychology, Georgia Institute of Technology, Atlanta, GA, United States

**Keywords:** executive function, cognitive flexibility, Wisconsin card sort task, scientific reasoning, science achievement

## Abstract

Cognitive flexibility as measured by the Wisconsin Card Sort Task (WCST) has long been associated with frontal lobe function. More recently, this construct has been associated with executive function (EF), which shares overlapping neural correlates. Here, we investigate the relationship between EF, cognitive flexibility, and science achievement in adolescents. This is important because there are fewer educational neuroscience studies of scientific reasoning than of other academically relevant forms of cognition (i.e., mathematical thinking and language understanding). Eighth grade students at a diverse middle school in the Midwestern US completed classroom-adapted measures of three EFs (shifting, inhibition, and updating) and the WCST. Science achievement was indexed by students’ standardized test scores and their end-of-the-year science class grades. Among the EF measures, updating was strongly predictive of science achievement. The association between cognitive flexibility and science achievement was comparatively weaker. These findings illuminate the relationship between EF, cognitive flexibility, and science achievement. A methodological contribution was the development of paper-and-pencil based versions of standard EF and cognitive flexibility measures suitable for classroom administration. We expect these materials to help support future classroom-based studies of EF and cognitive flexibility, and whether training these abilities in adolescent learners improves their science achievement.

## 1 Introduction

Executive function (EF) is a fundamental component of the human cognitive architecture. Individual differences in EF predict individual differences in complex cognitive abilities such as problem solving (Miyake et al., 2000). They also predict individual differences in important academic outcomes, most notably mathematical achievement (Van der Ven et al., 2013; Lee and Bull, 2016; Cragg et al., 2017) and reading ability (Christopher et al., 2012; Follmer, 2018; Georgiou et al., 2018). However, comparatively less is known about the relationship between EF and science achievement (St. Clair-Thompson and Gathercole, 2006; Gropen et al., 2011; St. Clair-Thompson et al., 2012; Rhodes et al., 2014, 2016; Bauer and Booth, 2019; Kim et al., 2021).

This is a critical gap because EF is potentially important for supporting core scientific reasoning abilities. For example, designing an experiment to evaluate a hypothesis requires systematically varying multiple hypothesis-relevant variables while controlling for or randomizing over hypothesis-irrelevant variables (Chen and Klahr, 1999; Klahr and Nigam, 2004; Kuhn et al., 2008). This top-down process makes heavy demands on attentional and working memory resources, which are limited and must be strategically managed. EF is also potentially relevant when a hypothesis is disconfirmed by new evidence (Popper, 1963), or more generally when science is seen as a competition between competing hypotheses (Duschl, 2020). As individuals work to coordinate their original theories or hypotheses with the evidence, disconfirmed hypotheses must be suppressed in working memory and new hypotheses capable of explaining the new results constructed.

The primary goal of the current study was to investigate the relationship between EF ability and science achievement. A secondary goal was to examine the relationship between cognitive flexibility and science achievement. Cognitive flexibility is commonly measured using the Wisconsin Card Sort Task (WCST; Grant and Berg, 1948; Eling et al., 2008). The secondary goal was motivated by the historical roots of EF in neuropsychological studies of the cognitive (in)flexibility of patients with frontal lobe damage (Milner, 1963; Shallice and Burgess, 1991; Miyake et al., 2000). Additionally, a prior study in the science education literature found that WCST performance was the single best predictor of scientific reasoning and science concept learning in middle and high school students (Kwon and Lawson, 2000).

### 1.1 Executive function and scientific reasoning

Following Miyake et al. (2000), we conceptualize EF as composed of three abilities. *Shifting* is the ability to switch between mental processes or representations when performing a task. *Inhibition* is the ability to suppress prepotent (i.e., typical or habitual) responses to stimuli in order to make novel responses. *Updating* is the ability to manage representations in working memory. Note that Miyake and colleagues have proposed alternate structures for EF in the ensuing years (e.g., Miyake and Friedman, 2012; Friedman and Miyake, 2017). However, the original analysis still dominates research on the relationship between EF and academic abilities such as language comprehension, mathematical thinking, and scientific reasoning, as reviewed below. We therefore adopt it here.

The cognitive and developmental psychology literatures give some reason to believe that EF might be related to science achievement. For example, when correct scientific theories are learned, they do not supplant incorrect beliefs; rather, incorrect beliefs persist and must be inhibited during future reasoning (Goldberg and Thompson-Schill, 2009; Kelemen and Rosset, 2009; Shtulman and Valcarcel, 2012; Knobe and Samuels, 2013). This suggests an important role for the inhibition EF in scientific reasoning (Mason and Zaccoletti, 2021). Another example, following Popper (1963), is that when facing disconfirming evidence, people might use inhibition to suppress the previous hypothesis. This frees limited working memory resources for constructing a new hypothesis. A final example is that inhibition, and attention more generally, might be important for students to remain engaged in science instruction and not fall behind (Gobert et al., 2015).

Prior developmental psychology studies have established a connection between EF and scientific reasoning. Composite EF ability positively predicts science learning in preschool children (Nayfield et al., 2013; Bauer and Booth, 2019). It also predicts knowledge of biological concepts in early elementary school children (Zaitchik et al., 2014; Tardiff et al., 2020). Finally, individual differences in inhibition predict science achievement in middle school children as measured by standardized science test scores (St. Clair-Thompson and Gathercole, 2006).

The current study extends these findings. It chooses measures of the three EFs based on their adaptability for whole-class administration. It first establishes that the EFs are separable. This sets the stage for the primary goal of investigating whether adolescents’ EF ability predicts science achievement as measured by standardized science test scores and by science class grades.

### 1.2 Cognitive flexibility, prefrontal cortex, and scientific reasoning

Executive function is a theoretical construct that distills and unifies multiple prior constructs. One of these is cognitive flexibility, a notion from cognitive neuropsychology and neuroscience that is often measured with the WCST task. This task requires evaluating a logical hypothesis against evidence, and when it is disconfirmed, shifting away from it and searching for an alternate hypothesis (Grant and Berg, 1948). Early studies established that WCST performance is impaired following lesions to prefrontal cortex (PFC; Milner, 1963; Shallice and Burgess, 1991). Such patients are unable to shift away from disconfirmed hypotheses, and instead cling to them, and as result make perseverative errors.

Prefrontal cortex is a key neural correlate of both EF and cognitive flexibility. Neuropsychology studies with lesion patients have shown the importance of PFC areas for supporting these cognitive abilities (Milner, 1963; Tsuchida and Fellows, 2013). Neuroimaging studies of adults and children have shown that the areas that comprise this region are active when people deploy the shifting, inhibition, and updating EFs (Miller and Cohen, 2001; Adleman et al., 2002; Durston et al., 2002; Tsujimoto, 2008), and also when they perform the WCST (Ríos et al., 2004; Lee et al., 2006; Nyhus and Barceló, 2009).

Prefrontal cortex is also active when people engage in logical reasoning (Goel, 2007; Prado et al., 2011), fluid reasoning (Lee et al., 2006; Cole et al., 2012), and, most relevantly, scientific reasoning. Fugelsang et al. (2005) found greater PFC activation when adults viewed animations depicting causal vs. non-causal events. Fugelsang and Dunbar (2005) provided adults with a scientific explanation that either did or did not provide a causal mechanism, and then showed them a sequence of experimental results that either disconfirmed or corroborated the explanation. Participants showed greater PFC activation when reasoning with a causal theory (vs. not), and when evaluating disconfirming (vs. corroborating) experimental evidence. In an instructional study, Mason and Just (2015) taught adults how four common mechanical devices (e.g., a bathroom scale) work. They found increased PFC activation when participants learned about the underlying causal mechanism and also when they learned how this mechanism achieved the function of the device.

A very different source of evidence for the relationship between WCST performance, PFC function, and scientific reasoning comes from a pioneering science education study by Kwon and Lawson (2000). They had middle and high school students complete measures of cognitive flexibility (WCST), problem solving (Tower of London task), and visuospatial reasoning (Group Embedded Figures task). They also administered the test of Lawson (1978), a standard measure of children’s scientific reasoning in the science education literature. Finally, they conducted an instructional study using a pre-post design where students learned about a novel science concept (air pressure) over 14 classroom lessons. WCST performance was the best single predictor—better even than chronological age—and explained 29% of the variance in scientific reasoning and 28% of the variance in pre-post gain in science concept learning.

The current study attempts to bring some unity to this set of findings. It adapts the WCST for whole-administration. It evaluates whether indices of WCST performance are related to which EFs. It also evaluates whether WCST performance predicts science achievement as measured by standardized science test scores and science class grades.

### 1.3 The current study

The primary research goal of the current study was to investigate the relationship between EF—conceptualized as shifting, inhibition, and updating—and science achievement in middle school students. It improves upon the only prior study to address this research goal, St. Clair-Thompson and Gathercole (2006), in three important ways. First, in the prior study, a clear shifting EF failed to emerge from the individual measures, and thus the relationship between that EF and science achievement could not be evaluated. This is an important gap because shifting is commonly thought to drive WCST performance (Miyake et al., 2000), and Kim et al. (2021) found shifting to predict science achievement in early elementary school children. To address this limitation, the current study employed a different set of tasks to measure the shifting EF. Second, St. Clair-Thompson and Gathercole (2006) did not directly predict science achievement from the inhibition and updating EFs, but rather from principal components extracted from a larger set of EF, working memory, and visuospatial measures. The current study isolated the predictive power of each EF by predicting science achievement directly from measures of shifting, inhibition, and updating ability. Third, St. Clair-Thompson and Gathercole (2006) used standardized test scores as their sole measure of science achievement. Standardized tests utilize restricted item formats that fail to capture authentic science practices such as making observations, executing experimental procedures, making sense of messy data, reasoning about causal connections and underling mechanisms, and writing up the results (Tolmie et al., 2016; Duschl, 2020). For this reason, the current study complemented standardized test scores with participants’ science class grades.

The secondary research goals concerned cognitive flexibility. Kwon and Lawson (2000) found the WCST to be the best predictor of scientific reasoning and science concept learning in middle (and high) school students. No subsequent study has attempted to replicate this result. The current study therefore examined the predictive relationship between WCST performance and science achievement. It also evaluated whether WCST performance is driven by the shifting EF in middle school students, as it is in adults (Miyake et al., 2000).

## 2 Methods

### 2.1 Participants

The participants were 110 eighth-grade students in five classrooms of a racially and ethnically diverse middle school in the Midwestern United States. Age information was not collected during the present study. Therefore, participant ages have been estimated using the ages of students currently enrolled in the teacher’s class, 13 years, 8 months. The race and ethnicity breakdown at the school level was 42.4% Hispanic or Latino, 1.0% American Indian or Alaska Native, 3.7% Asian, 13.3% Black or African-American, 28.5% White, and 11.0% two or more races. Parental consent and student assent were obtained in accordance with the University of Minnesota’s IRB. All students were in classes with the same science teacher. Parental consent forms were sent home with students and returned to the teacher. Students with signed parental consent were invited to assent to participate in the study. Those who did not have parental consent did not participate in the assent process. Students without parental consent or students who did not assent were given alternative activities by the teacher or could participate in the activities as a classroom activity and not have their work shared with the researchers. In this case, their work was discarded and not included in the analysis. Following the study activities, the teacher worked with the research team to debrief the students and discuss how each task measured how “their brain worked to process information.”

### 2.2 Design

The study utilized a correlational, individual differences design. Six EF measures and the WCST were administered over two class meetings with students in five class periods. The classroom teacher provided standardized test scores and classroom grades for the students who had parental consent and student assent. The science grades reflect performance on quizzes and tests covering content taught during the academic year.

### 2.3 Measures

#### 2.3.1 Executive function

Researchers have proposed multiple measures for each of the shifting, inhibition, and updating EFs in developmental samples (Huizinga et al., 2006; St. Clair-Thompson and Gathercole, 2006; Van der Ven et al., 2013; Brydges et al., 2014a; Bauer and Booth, 2019; Kim et al., 2021). This study utilized two tasks to measure each EF. The tasks were chosen based on their usage in the seminal study of Miyake et al. (2000), their effectiveness in prior studies of middle-school students, and their potential adaptability for whole-class administration (Huizinga et al., 2006; St. Clair-Thompson and Gathercole, 2006; Van Boekel et al., 2017; Varma et al., 2018). Multiple tasks include completion time as a dependent variable. Completion time was self-reported by participating students. To facilitate a whole-class administration, a digital timer was projected on a screen in the front of the classroom. Students were instructed to write down the time on their sheets when they completed a timed task. Multiple members of the research team walked around the classroom in order to closely monitor the class and remind students to record the time when they completed the timed tasks.

**Figure 1:**
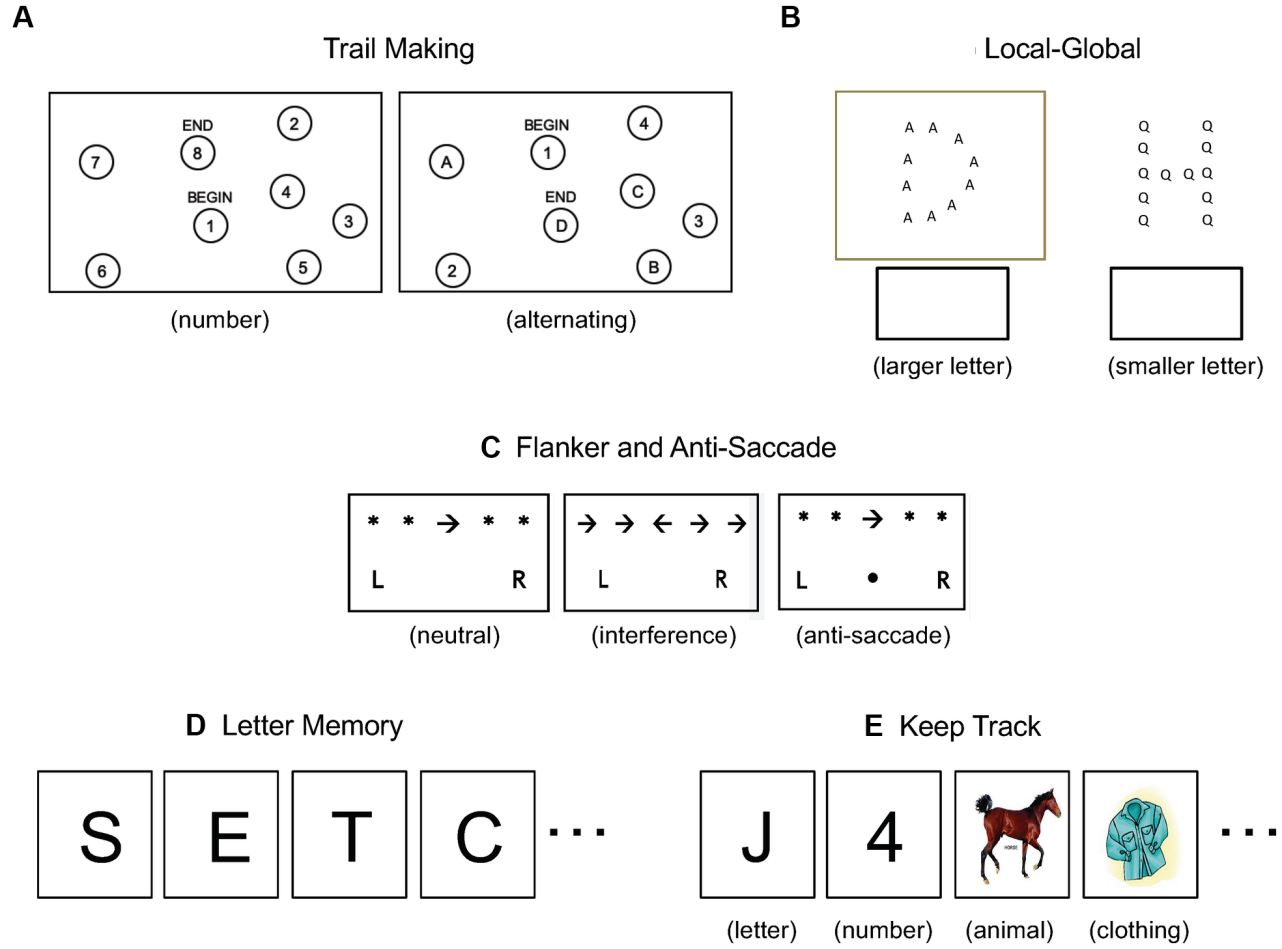
Example stimuli for the shifting tasks **(A,B)**, the inhibition tasks **(C)**, and the updating tasks **(D,E)**.

Shifting was measured using the trail-making and local–global tasks. For the trail-making task, participants completed two sets of “connect the dots” sheets. For the number sheets, they connected scattered dots labeled 1–25 in ascending order. For the alternating sheets, they connected scattered dots labeled 1–13 and A-L in alternating and ascending order: 1-A-2-B- and so on. See Figure 1A for reduced examples of the sheets. After completing each set, they consulted a timer projected at the front of the classroom and recorded their completion time. The dependent variable was completion time on the alternating sheets minus completion time on the number sheets. For the local–global task, participants were given a packet of 90 stimuli. Each stimulus consisted of a larger letter (e.g., “G”) composed of smaller letters (e.g., “A”). If the stimulus was boxed, participants identified the larger letter; otherwise they identified the smaller letter. See Figure 1B for examples of boxed and unboxed stimuli. The dependent variable was the number of correct identifications made in 2 min (90 maximum).

Inhibition was measured using the flanker and anti-saccade tasks, which were implemented in a three-page packet. The first page contained 32 neutral stimuli. Each stimulus consisted of a central arrow flanked by asterisks (e.g., “* * → * *”) and participants indicated the arrow’s direction by circling “L” or “R.” The second page contained 32 flanker stimuli. For half, the central arrow was flanked by arrows pointing in the same direction (e.g., “**→ → → → →**”), whereas for the other 16 (interference) stimuli, the flanking arrows were pointing in the opposite direction (e.g., “**→ → ← → →**”). The task was again to indicate the direction of the central arrow by circling “L” or “R.” The third page contained 32 anti-saccade stimuli. For half, a central arrow was flanked by asterisks (e.g., “* * ← * *”) and participants indicated its direction by circling “L” or “R.” For the other half, a dot appeared between the “L” and “R,” signaling that the task was to indicate the direction *opposite* to that of the central arrow. See Figure 1C for example neutral, interference, and anti-saccade stimuli. After completing each page, participants consulted a timer projected at the front of the classroom and recorded their completion time. The dependent variable for the flanker task was completion time on the interference sheet minus the neutral sheet; for the anti-saccade task, it was completion time on the anti-saccade sheet minus the neutral sheet.

Updating was measured using the letter memory and keep track tasks. For the letter memory task, participants viewed eight sequences of letters, two each of lengths 5, 7, 9, and 11 letters. For each sequence, the letters were projected at the front of the classroom one at a time, with the last letter followed by a recall cue. See Figure 1D for example stimuli. Upon seeing the cue, participants recorded the last four letters of the sequence on their response sheet. The dependent variable was the total number of correct letters (8 × 4 = 32 maximum). The keep track task was similar to the letter memory task. Participants viewed four sequences of numbers, letters, and animals followed by two sequences that additionally included a fourth category, clothing items. See Figure 1E for examples of each category. Each sequence consisted of 15 items that were projected at the front of the classroom one at a time and immediately followed by a recall cue. Upon seeing the cue, participants recorded the last instance of each category on their response sheet. The dependent variable was the total number of correct instances (4 × 3 + 2 × 4 = 20 maximum).

#### 2.3.2 Wisconsin card sort task

**Figure 2:**
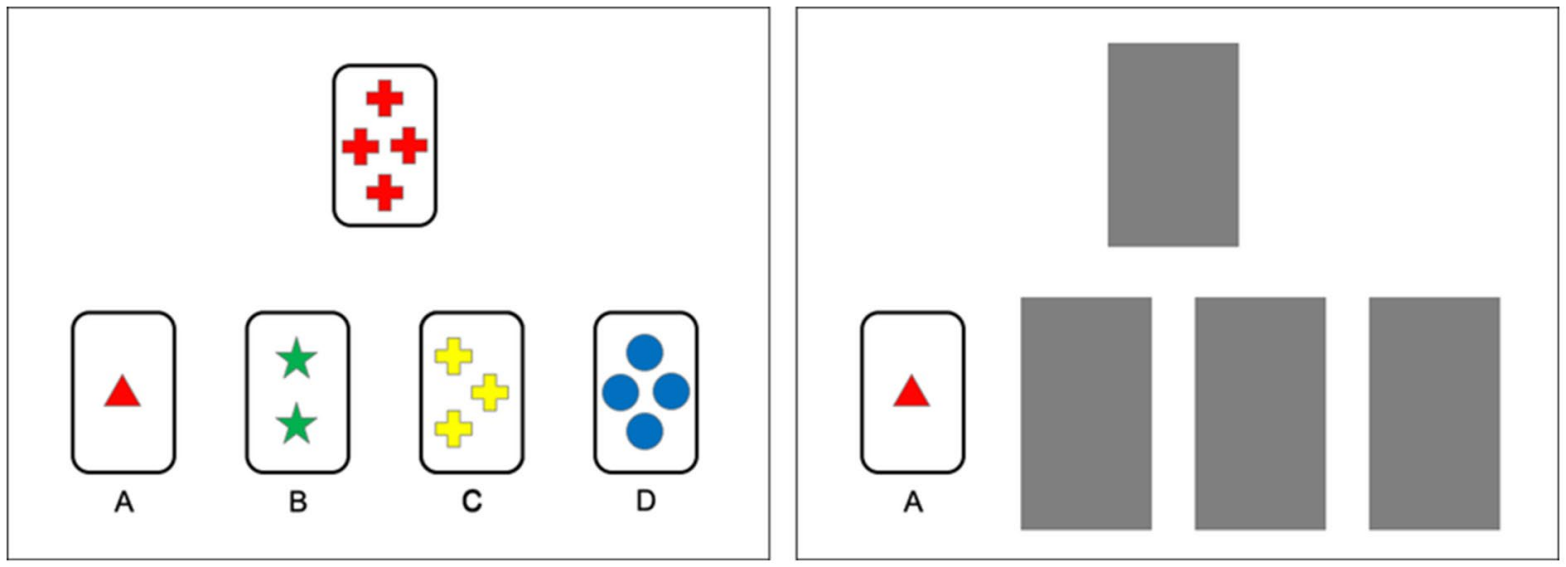
Example stimulus presentation (left) and feedback presentation (right) for the WCST.

We measured cognitive flexibility using a version of the WCST shortened and adapted for whole-class administration (Varma et al., 2016, 2018). Participants viewed 48 stimuli one at a time. Each stimulus showed one target card, which changed for each stimulus, and four standard cards labeled A–D, which remained the same. Each card varied in color, number, and shape (four levels of each attribute). Participants were asked to judge which standard card the target card “was most similar to” based on an unknown rule by writing down A–D on their response sheet. Feedback was then provided by masking all of the standard cards but the correct one. See Figure 2 for an example of a stimulus and masked feedback. Critically, the correct rule changed every eight cards, cycling twice through “same color,” “same number,” and “same shape.”

Of importance is how participants respond to the *second* stimulus following a rule change. For the first stimulus following a rule change, participants generally applied the previous rule and received feedback that their judgment was incorrect. This incorrect response should be surprising to the participant. The second stimulus is the first opportunity for them to respond to the surprising, disconfirming evidence by deciding to shift to a new rule, choosing one, and applying it. We coded participant responses into three dependent variables: number of perseverative errors (i.e., incorrectly applying the previous, disconfirmed rule, resulting in an error), number of systematic hits (i.e., properly shifting to a new rule, and by good luck choosing the one that produces the correct judgment), and number of systematic errors (i.e., properly shifting to a new rule, but by bad luck choosing the one that produces an incorrect judgment). The first variable is the classic index of errorful performance on the WCST (Grant and Berg, 1948; Heaton et al., 2004) and is associated with the shifting EF in adults (Miyake et al., 2000). The other variables do not index errorful performance, but rather organized exploration of a new rule following disconfirmation of the previous rule. They reflect the processes by which scientists are trained to respond to disconfirming evidence: by dismissing the previous hypothesis (Popper, 1963) and systematically searching for a new one (Klahr and Dunbar, 1988).

#### 2.3.3 Scientific achievement

Two measures of scientific achievement were collected for all participants: scores on the standardized science achievement test taken by all eighth graders in the state at the end of the school year, and final grades in their science class.

### 2.4 Procedure

The six EF tasks and the WCST were administered over two class meetings, each lasting 50 min, in a whole-class fashion. At the end of the school year, participants’ standardized science achievement test scores and final science class grades were obtained from the classroom teacher.

## 3 Results

Absences and class interruptions (e.g., a fire alarm during one class period), prevented some students from completing all measures. Therefore, each analysis concerned only a subset of the sample. Table 1 provides the descriptive statistics for all measures.

**Table 1:** Descriptive statistics for all analyzed measures.

Measure	*N*	*Mdn*	*M*	*SD*	*Min*	*Max*
Executive function						
Shifting.z	68		−0.06	0.53	−1.40	1.45
Inhibition.z	92		0	0.88	−1.66	2.87
Updating.z	84		0.01	0.84	−2.34	1.37
WCST						
Systematic hits	81	3	3.02	1.53	0	5
Systematic errors	81	1	1.07	1.03	0	4
Perseverative errors	81	2	3.48	3.55	0	13
Science achievement						
Standardized score	108	848	847	14	803	881
Final grades	109	7	7.29	3.79	0	12

For the descriptive statistics for all component executive function measures (raw and standardized), see Supplementary Table S1.

### 3.1 EF abilities and their separability

The two shifting measures were correlated [*r*(66) = −0.440, *p* < 0.001], as were the two inhibition measures [*r*(90) = 0.561, *p* < 0.001] and the two updating measures [*r*(82) = 0.512, *p* < 0.001].

To evaluate the separability of the three EFs, we conducted a principal components analysis of the six measures, with varimax rotation; see Table 2.

**Table 2:** Principal components analysis of the EF measures after varimax rotation.

EF	Task	PC 1	PC 2	PC 3	PC 4
Shifting	Trail-Making	0.283	−0.247	−0.155	0.883
	Local–Global	−0.234	0.116	0.948	−0.137
Inhibition	Flanker	0.815	−0.109	−0.217	0.347
	Anti-Saccade	0.918	−0.049	−0.151	0.070
Updating	Letter memory	−0.345	0.825	0.275	−0.004
	Keep track	0.127	0.845	−0.049	−0.360
Eigen value		2.898	1.250	0.683	0.556
% Variance		48.30%	20.82%	11.38%	9.26%

Two components had eigenvalues greater than 1. The inhibition measures loaded on the first component and the updating measures on the second component. Because we had theoretical reasons to expect a shifting factor, we looked at additional components. One shifting measure (local–global) loaded on the third component and the other (trail-making) on the fourth component; together, these components accounted for roughly as much variance (20.64%) as the second component (20.82%). Thus, the principal components analysis found clear evidence for the inhibition and updating EFs, and some evidence for the shifting EF.

A composite measure of inhibition was computed for each participant by converting their performance on the flanker and anti-saccade tasks to *z*-scores and averaging them. Composite measures of updating and shifting were computed analogously. These composite measures were used below to predict science achievement and WCST performance.

### 3.2 EF ability predictors of science achievement

The primary research goal was to investigate whether and how EF predicts science achievement. Consider the first measure of science achievement, standardized science achievement test scores. Table 3 shows the correlations between this measure and the three EFs. Significant correlations were observed with inhibition [*r*(91) = −0.280, *p* = 0.007] and updating [*r*(83) = 0.503, *p* < 0.001]. To better understand the inter-relationships among the three EFs, they served as predictor variables in a regression analysis with standardized science achievement test scores as the criterion variable; see Table 4. The regression model explained 28.5% of the variance in standardized science achievement test scores, which was significant [*F*(3, 55) = 7.293, *p* < 0.001]. Only the updating variable was a significant predictor ( β = 0.501, *t* = 4.194*, p* < 0.001).

**Table 3:** Correlations of EF abilities and WCST performance indices to the science achievement measures.

		Standardized test scores		Science class grades	
		*r*	*p*	*r*	*p*
EF	Shifting	−0.122	0.320	−0.139	0.259
	Inhibition	−0.280	0.007	−0.145	0.167
	Updating	0.503	< 0.001	0.538	< 0.001
WCST	Perseverative errors	−0.410	< 0.001	−0.418	< 0.001
	Systematic hits	0.433	< 0.001	0.459	< 0.001
	Systematic errors	−0.176	0.118	−0.296	0.008

**Table 4:** Regressions predicting the science achievement criterion variables from EF abilities and from WCST performance indices.

		Standardized test scores			Science class grades		
		β	*t*	*p*	β	*t*	*p*
EF	Shifting	−0.125	−1.087	0.282	−0.107	−0.921	0.361
	Inhibition	−0.017	−0.143	0.886	0.124	1.026	0.309
	Updating	0.501	4.194	< 0.001	0.510	4.206	< 0.001
WCST	Perseverative errors	−0.209	−1.498	0.138	−0.255	−1.862	0.067
	Systematic hits	0.329	1.937	0.056	0.225	1.346	0.182
	systematic errors	0.050	0.376	0.708	−0.124	−0.946	0.347

Next, consider the second measure of science achievement, science class grades. Table 3 shows the correlation between this measure and the three EFs. The only significant correlation was with updating [*r*(83) = 0.538, *p* < 0.001]. A regression model fit with the three EFs as predictor variables and science class grades as the criterion variable explained 26.4% of the variance, which was significant [*F*(3, 55) = 6.583, *p* = 0.001]; see Table 4. Again, only the updating variable was a significant predictor ( β = 0.510, *t* = 4.206*, p* < 0.001).

### 3.3 WCST performance and its relationship to EF abilities and science achievement

Among the three indices of WCST performance, number of perseverative errors and number of systematic hits were correlated [*r*(88) = −0.613, *p* < 0.001], as were number of systematic hits and number of systematic errors [*r*(88) = −0.566, *p* < 0.001]. Number of perseverative errors and number of systematic errors were uncorrelated [*r*(88) = 0.191, *p* = 0.072].

One secondary research goal was to investigate the relationship between EF ability and WCST performance (reflecting cognitive flexibility). We therefore conducted three regressions, each predicting an index of WCST performance from the three EFs. The results are shown in Table 6.

First, consider number of perseverative errors, the standard index of WCST performance in the literature. The regression model explained 14.0% of the variance in this criterion variable, which was marginally significant [*F*(3, 49) = 2.668, *p* = 0.058]. Only the shifting variable was a significant predictor ( β = 0.272, *t* = 2.041*, p* = 0.047), a finding consistent with the Miyake et al. (2000) study of adults. Note that the updating variable was a marginally significant predictor (*p* = 0.099), which is consistent with the Huizinga et al. (2006) study of middle- and high-school children.

Next, consider number of systematic hits, which reflects the successful search for a new rule following disconfirmation of the previous rule. The regression model explained 21.0% of the variance in this criterion variable, which was significant [*F*(3, 49) = 4.345, *p* = 0.009]. Only the updating variable was a significant predictor ( β = 0.445, *t* = 3.346*, p* = 0.002).

Finally, consider the number of systematic errors, which also reflects the search for a new rule following disconfirmation of the previous rule, but in this case reflects an unsuccessful search. The regression model explained 11.3% of the variance, which was not significant [*F*(3, 49) = 2.082, *p* = 0.115]. Only the inhibition variable approached significance ( β = 0.270, *t* = 1.924, *p* = 0.060).

Another secondary research goal was to investigate whether cognitive flexibility predicts science achievement. We first focused on standardized science achievement test scores. As Table 3 shows, these scores were significantly correlated with perseverative errors [*r*(79) = −0.410, *p* < 0.001] and systematic hits [*r*(79) = 0.433, *p* < 0.001]. A multiple regression analysis revealed that 21.9% of the variance in science scores was predicted by the three WCST variables, which was significant [*F*(3, 76) = 7.120, *p* < 0.001]; see Table 4.

The second measure of science achievement, science class grades, was significantly correlated with all three WCST variables: preservative errors [*r*(79) = −0.418, *p* < 0.001], systematic hits [*r*(79) = 0.459, *p* < 0.001], and systematic errors [*r*(79) = −0.296, *p* = 0.008]; see Table 3. A multiple regression analysis found that 24.6% of the variance in science class grades was predicted by the three WCST variables, which was significant [*F*(3, 76) = 8.284, *p* < 0.001]; see Table 4.

## 4 Discussion

This study investigated the relationship between EF, cognitive flexibility, and science achievement in middle school students. For each EF—shifting, inhibition, and updating—there were two measures, adapted for classroom administration. The two measures were highly correlated. A principal components analysis found strong evidence for inhibition and updating components, and weaker evidence for a shifting component, consistent with a unity and diversity of executive functions (Miyake et al., 2000). This licensed computing composite measures of each EF ability and addressing the primary and secondary research goals.

The primary research goal was to investigate whether and how EF abilities predict science achievement. The central finding was that only the updating EF was a consistent and significant predictor of science achievement, whether measured using standardized science achievement scores or science class grades. This finding can be understood relative to the findings and limitations of the only prior study of the relationship between EF and science achievement. St. Clair-Thompson and Gathercole (2006) found only the updating to be a significant predictor of standardized science achievement scores, which the current study replicated. A limitation of this earlier study was that a clear shifting component failed to emerge from the principal components analysis, and it was therefore unable to evaluate the relationship between this EF and standardized science achievement scores. A clearer shifting EF emerged in the current study, presumably because it utilized a different and more varied set of shifting tasks. That shifting failed to correlate with standardized science achievement scores in the current study is important new information. St. Clair-Thompson and Gathercole (2006) collected only one measure of science achievement, standardized science achievement test scores. Such tests employ restricted item formats and artificial time limits, and do not capture the richness of authentic science practices. For this reason, the current study collected a second measure of science achievement, science class grades. That updating was the only significant predictor of this richer measure of science achievement is also important new information.

The secondary research goals concerned the relationship of cognitive flexibility as measured by the WCST to science achievement. The WCST is important for two reasons. First, Kwon and Lawson (2000) found WCST performance to be the best predictor of scientific reasoning and science concept learning in middle (and high) school students – better than problem solving, visuospatial reasoning, and chronological age. No subsequent studies have attempted to replicate this potentially important result. Second, the standard index of WCST performance, number of perseverative errors, is associated with the shifting EF in adults (Miyake et al., 2000). Shifting has a ready analog in scientific reasoning: when facing disconfirming evidence, perseverative errors can be conceptually mapped to failing to dismiss the falsified hypothesis. However, this is just one of several potentially relevant indices of WCST performance. The other indices considered here, number of systematic hits and number of systematic errors, can be conceptually mapped to, following falsification of the current hypothesis, searching for a new hypothesis. It is an open question which EFs are associated with these other indices of WCST performance. The current study addressed these gaps.

With respect to the relationship between the WCST and EF abilities, number of preservative errors was predicted by the shifting EF, as in prior studies of adults (Miyake et al., 2000). It was also marginally (*p* = 0.099) predicted by updating ability, consistent with the finding of an association between number of perseverative errors and working memory capacity in a prior study of middle school students (Huizinga et al., 2006). Note that number of perseverative errors was *not* predicted by inhibition ability, in contrast to the significant correlation between the two variables observed by Brydges et al. (2014b). It should be noted that the participants in that study were younger (9-year-old) than those of the current study, the observed correlation was small in size (*r* = 0.23, *p* < 0.05), and the analyses did not simultaneously control for the other EFs as was done here and in Miyake et al. (2000) and Huizinga et al. (2006). Finally, of the other indices of WCST performance considered in the current study, number of systematic hits was predicted by updating ability. In addition, number of systematic errors was marginally (*p* = 0.060) predicted by inhibition ability.

With respect to the relationship between WCST performance and science achievement, the classic measure, number of perseverative errors, predicted standardized science achievement scores, but only marginally (*p* = 0.067). This represents a weak replication of Kwon and Lawson (2000). Of the newer measures, number of systematic errors predicted science achievement, but again only marginally (*p* = 0.056).

There are a number of limitations of the current study. Most notable was the relatively small sample size. Replicating the current study with a larger sample might clarify some of the marginal findings for the WCST. Below, we focus on the theoretical implications and limitations of the current study, and use these to identify directions for future research.

### 4.1 Measuring EF

Researchers have proposed multiple measures of each EF ability. Table 5 lists those used in the seminal study of EF in adults of Miyake et al. (2000) and in four studies of EF in middle school students that closely followed the Miyake decomposition (Huizinga et al., 2006; St. Clair-Thompson and Gathercole, 2006; Brydges et al., 2014a). All prior studies administered EF measures using computer-based implementations, and all tested participants individually. A key methodological innovation of the current study was the development of new versions of classic measures of shifting, inhibition, and updating that can be administered using paper-based materials to participants tested in groups. These materials are more appropriate for classroom-based research.

**Table 5:** Executive function tasks used in Miyake et al. (2000) and in studies of adolescents.

Study	Population	Administration	Shifting	Inhibition	Updating
Miyake et al. (2000)	Adults	Individual; computer	Local–global	Anti-saccade	Keep track
			Plus-minus	Stop-signal	Letter memory
			Number-letter	Stroop	Tone monitoring
Huizinga et al. (2006)	Children (elementary, middle, and high)	Individual; computer	Local–global	Flanker	Running memory
			Dots-triangles	Stop-signal	Mental counters
			Smiling faces	Stroop	Tic tac toe
St. Clair-Thompson and Gathercole (2006)	Children (middle)	Individual; computer	Local–global	Stop-signal	Keep track
			Plus-minus	Stroop	Letter memory
van der Ven et al. (2013)	Children (elementary)	Individual; computer	Trail-making	Animal Stroop	Keep track
			Animal shifting	Local–global	Digit span backwards
			Sorting task	Simon task	Odd one out
					Keep track
Brydges et al. (2014b)	Children (elementary, middle)	Individual; psychometric test kit and computer	WCST	Stroop	Digit span backwards
			Semantic fluency	Go/no-go	Sentence repetition
			Letter monitoring	Compatibility RT	Letter-number sequencing
Current study	Children (middle)	Group; paper-and-pencil	Local–global	Anti-saccade	Keep track
			Trail-making	Flanker	Letter memory

The design of these measures merits further comment. We initially selected a superset of EF tasks for adaptation subject to the following criteria:

- be paper-based;
- be group-administered;
- have a speed or memory challenge that student participants find motivating; and
- allow minimal opportunities for cheating

This required multiple cycles of designing and re-designing paper-based versions of EF tasks, piloting with the research team, and piloting with middle school students. Several tasks were abandoned when they could not be refined to meet the criteria above. This process extended over several years in an ongoing project framed as the “Brain Olympics” (Varma et al., 2016). The students who participated in the study enjoyed competing to finish each “event” as quickly as possible and/or remember as much information as possible.

The correlational and principal components analyses demonstrate that the six measures are a viable foundation for future classroom-based research on the relationship between EF ability and academic achievement. Of course, the current task selections and paper-based implementations are not final, and will benefit from further refinement in future research. The distal goal is to identify a canonical set of tasks for measuring shifting, inhibition, and updating in middle school students.

A more proximal goal is to develop a better set of shifting measures. In the only other study to investigate the relationship between EF and science achievement, St. Clair-Thompson and Gathercole (2006) measured shifting using the local–global and plus-minus tasks; see Table 5. These tasks were uncorrelated with each other (*r* = 0.13, *p* > 0.05), and they failed to load on a single principal component. Instead, the local–global task loaded on a component driven by inhibition tasks and the plus-minus task loaded on one driven by updating tasks. For this reason, the authors excluded the shifting tasks from subsequent analyses in their study, and did not attempt to relate the shifting EF to science achievement. The current study retained the local–global task, in part because many prior studies also included it (see Table 5). For the other shifting task, a review of the neuropsychological literature for tasks sensitive to PFC lesions revealed that the trail making task best met criteria (a) through (d). These two shifting measures offered a partial improvement on St. Clair-Thompson and Gathercole (2006). They were correlated with each other (*r* = −0.440, *p* < 0.001), and they did not load on the inhibition and updating components in the PCA. However, they did not both load on the same component. Instead, they loaded separately on the third and fourth components, and these together accounted for significant variance (20.64%). This was sufficient to license computing a composite shifting EF and analyzing its relationship to science achievement (and to indices of WCST performance). Because this improvement on St. Clair-Thompson and Gathercole (2006) was only partial, a goal for future research is to identify a more coherent set of shifting tasks. One problem with the trail making task is that it may not be a pure measure of shifting because it also requires significant visuospatial processing.

**Table 6:** Regressions predicting each WCST performance index from the three EF abilities.

Criterion variable	Predictor variable	β	*t*	*p*
Perseverative errors	Shifting	0.272	2.041	0.047
	Inhibition	−0.122	−0.883	0.382
	Updating	−0.234	−1.682	0.099
Systematic hits	Shifting	−0.097	−0.763	0.449
	Inhibition	0.036	0.275	0.785
	Updating	0.445	3.346	0.002
Systematic errors	Shifting	−0.001	−0.008	0.994
	Inhibition	0.270	1.924	0.060
	Updating	−0.139	−0.988	0.328

### 4.2 Predicting science achievement

The current study investigated whether EF abilities and indices of WCST performance predict science achievement. A novel feature was the utilization of two measures of science achievement. Following St. Clair-Thompson and Gathercole (2006), we collected participants’ science achievement scores on a standardized test administered to all students in the state. We also collected students’ science class grades, which reflect their mastery of authentic science practices too complex to measure using the limited item formats of standardized tests. Regression analyses revealed that of the multiple EF and WCST predictors, only updating was significantly associated with either measure of science achievement; in fact, it was associated with both.

This result was both expected and surprising. It was expected in replicating the finding of St. Clair-Thompson and Gathercole (2006) that updating was the only EF ability to predict standardized science achievement scores in middle school students. It was also expected in replicating the absence of an association between either the inhibition or shifting EF and science class grades observed by Rhodes et al. (2014, 2016) in middle school students. It was surprising in failing to replicate the finding of Kwon and Lawson (2000) that number of perseverative errors on the WCST was the best predictor (among several measures of frontal lobe function) of scientific reasoning ability and science concept learning. Perseverative errors are commonly thought to reflect failings of the shifting EF (Miyake et al., 2000), and yet neither perseverative errors nor shifting ability predicted science achievement in the current study.

Why might updating be the most important EF for predicting science achievement? Recall that updating is the ability to manage representations in working memory (WM). This raises the possibility that WM capacity may perhaps be an even better predictor of science achievement. The letter memory and keep track tasks used to measure updating in the current study (and in prior studies—see Table 5) require people to process representations in WM in a very artificial manner. In both tasks, participants monitor a sequence of stimuli, compare each one to the representations in WM, and occasionally replace one of those representations with an encoding of the new stimulus. By contrast, measures of WM capacity such as the reading span task (Daneman and Carpenter, 1980) and the operations span task (Turner and Engle, 1989) require participants to process representations in WM in a richer manner, by parsing sentences or performing arithmetic calculations, respectively. This is important because WM capacity is defined as the ability to both store and process information (Baddeley and Hitch, 1974; Daneman and Carpenter, 1980).

Prior research has demonstrated that WM capacity is a good predictor—perhaps the best single predictor—of verbal ability, mathematical ability, and other cognitive abilities (Jarvis and Gathercole, 2003; Yuan et al., 2006; Christopher et al., 2012; Cragg et al., 2017; Follmer, 2018). Thus, the current finding that updating predicts science achievement suggests that WM capacity as measured by complex span tasks might be an even better predictor. The evidence for this prediction is both sparse and mixed. St. Clair-Thompson and Gathercole (2006) found that WM capacity predicted standardized science achievement scores in middle school students, and Rhodes et al. (2014, 2016) found that it predicted their science class grades. By contrast, St. Clair-Thompson et al. (2012) found no association between WM capacity and science achievement. However, that study did not employ complex span measures of WM (Daneman and Carpenter, 1980; Turner and Engle, 1989). Instead, it utilized simpler measures aligned with the three components of the model of Baddeley and Hitch (1974); it also considered an older population and science achievement was indexed by United Kingdom A-level grades. Thus, the question of whether WM capacity predicts science achievement remains open.

Note that the failure of the shifting and inhibition EFs and the three indices of WCST performance to emerge as significant predictors of science achievement should not be understood as final. Indeed, a number of these measures correlated significantly with science achievement; see Table 3. It was only in the larger regression models that they failed to contribute significant, unique predictive power. Future studies employing different measures of these EFs and of cognitive flexibility might yield more refined results.

### 4.3 Toward a training study

The finding of an association between the updating EF and science achievement points the way to future research investigating the causality of this relationship. Such evidence could come from a training study. Does training the updating ability produce increases in children’s science achievement? This question belongs to the larger effort to establish whether training EF and WM lead to improvements in other cognitive abilities such as language comprehension (e.g., Chein and Morrison, 2010), mathematical thinking (e.g., Holmes et al., 2009), fluid reasoning (Jaeggi et al., 2008; Au et al., 2014), and creative thinking (Lin et al., 2018). There are reasons to believe that training EF or WM might improve science achievement. Both are important for the basic mechanisms of scientific reasoning as documented by researchers (e.g., Jarvis and Gathercole, 2003; St. Clair-Thompson and Gathercole, 2006; Yuan et al., 2006; St. Clair-Thompson et al., 2012; Rhodes et al., 2014, 2016; Varma et al., 2018), which are in turn important for the scientific practices emphasized by the Next Generation Science Standards (NGSS Lead States, 2013). These practices include designing and conducting scientific investigations; using appropriate techniques to analyze and interpret data; developing descriptions, explanations, predictions, and models using evidence; and thinking critically and logically to construct the relationships between evidence and explanations.

The most direct approach would be to train participants on EF or WM tasks and to measure the effect on science achievement and science class grades. An approach more amenable to implementation in science classrooms would be to train participants by having them play games that target these abilities. Playing experimenter-designed board games and computer games would potentially be engaging for students (Sinatra et al., 2015). These activities have been shown to improve children’s numerical abilities, and ultimately their mathematical achievement (Ramani and Siegler, 2008; Butterworth et al., 2011). Commercial games with particular game mechanics have been shown to exercise computational thinking in players (Berland and Lee, 2011). A training study that utilizes experimenter-designed or commercial games might be effective for improving scientific thinking and science achievement (Homer and Plass, 2014).

## Data availability statement

The raw data supporting the conclusions of this article will be made available by the authors, without undue reservation.

## Ethics statement

The studies involving humans were approved by University of Minnesota Institutional Review Board. The studies were conducted in accordance with the local legislation and institutional requirements. Written informed consent for participation in this study was provided by the participants’ legal guardians/next of kin.

## Author contributions

KV, MB, GA, and SV contributed to conception and design of the study. KV and SV organized the database and performed the statistical analysis. KV wrote the first draft of the manuscript. KV, MB, and SV wrote the sections of the manuscript. All authors contributed to the article and approved the submitted version.
