# Supplementary material for: Executive function predictors of science achievement in middle-school students
Source: Front Psychol. 2023 Nov 28;14:1197002. doi: 10.3389/fpsyg.2023.1197002 (PMC10713847; doi:10.3389/fpsyg.2023.1197002)
Supplement: Supplementary file 1 [file Data_Sheet_1.PDF]

Supplementary Material

Executive function predictors of science achievement in middle-school students

Keisha Varma* , Martin Van Boekel, Gary Aylward, and Sashank Varma

*** Correspondence:** Keisha Varma: keisha@umn.edu

1 Supplementary Table

Table S1. Descriptive statistics for all component executive function measures (raw and standardized), as well as the three composite EF variables.

Measure	*N*	*Mdn*	*M*	*SD*	*Min*	*Max*
Raw component EF measures						
TM.A	76	53.5	60.54	21.91	26	126
TM.B	75	89	95.47	42.94	31	260
InhibCon	92	32	32.65	8.82	18	70
Flanker	92	38	40.64	13.37	20	119
AntiSaccad	92	49	49.66	12.78	27	99
LM.items	85	23	23.31	4.33	13	32
KT.items.3	96	11	10.25	2.55	0	12
KT.items.4	96	6	5.59	2.03	0	8
LG.corr	90	67	65.21	17.48	18	90
LG.comp	90	69.5	67.13	15.80	32	90
Standardized component EF measures						
TM.B.z	75		0.00	1.00	-1.50	3.83
LG.corr.z	90		0.00	1.00	-2.70	1.42
Flanker.z	92		0.00	1.00	-1.54	5.86
AntiSaccad.z	92		0.00	1.00	-1.77	3.86
LM.items.z	85		0.00	1.00	-2.38	2.01
KT.items.4.z	96		0.00	1.00	-2.76	1.19
Composite EF measures						
shift.z	68		-0.06	0.53	-1.40	1.45
inhib.z	92		0.00	0.88	-1.66	2.87
update.z	84		0.01	0.84	-2.34	1.37
